# Burns demand our attention

**DOI:** 10.4103/0970-0358.70713

**Published:** 2010-09

**Authors:** Prabhat Shrivastava, Sunil Keswani

**Affiliations:** Department of Burns and Plastic Surgery, Lok Nayak Hospital and Associated Maulana Azad Medical College, New Delhi – 110 002, India. E-mail: drprabhat@rediffmail.com; 1 National Burns Centre, Sector 12, Airoli, Navi Mumbai – 400 708, India. E-mail: smkeswani@gmail.com

An extensive burn injury is one of the most devastating conditions encountered in medicine. It severely impacts the physical, physiological, psychological, social and financial status of not only the victim but that of his entire family. Better understanding of pathophysiology, early burn wound excision and provision of skin cover, development of newer skin substitutes and equivalents, and remarkably improved anaesthetic procedures, ICU care and ventilation have surely contributed to better survival statistics of a thermally injured individual. But despite so many advances, the ultimate goal of making a burn victim functionally independent and aesthetically acceptable to achieve the societal reintegration and return to profession still remains a long cherished but elusive goal for majority of the burn survivors even in the developed world.

Statistics prove that developing countries have a huge load of burn injuries (estimated 6–7 million new cases per year in India) with majority of thermally injured victims being primarily managed in the peripheral hospitals. Private sector does not have many established and functioning burn units. Very few can afford the exorbitant cost of treatment at the corporate set up. Moreover, a large number of victims are from low socioeconomic status and are not insured either. The National Programme for Prevention of Burn Injuries is also not yet in place in our country.

In India, meagerly equipped government hospitals bear the brunt of this massive load. At many places, even the services of all the members of the multidisciplinary team are also not available. Despite this entire crunch, such a massive load has been managed for years by plastic surgeons and general surgeons of these government hospitals. Besides the surgeon, the credit must also go to the selfless and dedicated service of the staff, especially the resident doctors and the nursing personnel. These overworked and underappreciated vital cogs are intimately associated with the day to day care of these patients in the casualty, burn wards, OTs, OPD, physiotherapy, etc. Staying abreast with the recent trends simultaneously keeping basic fundamentals firmly in place is essential for all the members of the burn team. The dedicated manuals/texts/journals play a major role in this aspect.

This special supplement of *Indian Journal of Plastic Surgery* (IJPS) is aimed for this very purpose. A broad range of topics have been chosen for this issue. We shall remain indebted to our national and overseas contributors, all of whom have loads of experience and are leaders in their respective fields. They have done a magnificent job of bringing together the most current information regarding various aspects of burn management. The reading of this volume makes a comprehensive review of the subject and we are hopeful that it shall be useful for the residents and the teachers alike.

In recent times, aesthetic surgery has taken such a lead in the minds of young plastic surgeons that burn management has been literally overlooked as a discreet topic. ‘Burn care’, despite being such a vast specialty within the super specialty of Plastic Surgery, is assigned just one session in the four-and-a-half day long National Plastic Surgery meetings! In this era of highly lucrative cosmetic surgery, the editorial staff at IJPS needs to be complimented for having dedicated the entire issue to the cause of burns.

Wish you all a ‘happy and pleasant’ reading.

